# Transcriptomic Identification of Candidate Biomarkers Associated With Vascular Calcification‐Related Signaling Pathways in Immunoglobulin A Nephropathy

**DOI:** 10.1155/ijog/3008549

**Published:** 2026-11-05

**Authors:** LiJuan Cheng, ZiJin Xin, XinLi Zhao, JingFeng Wang

**Affiliations:** ^1^ Department of Nephrology and Immunology Shenzhen Qianhai Shekou Free Trade Zone Hospital Shenzhen China skhosp.cn; ^2^ School of Pharmaceutical Business Guangdong Pharmaceutical University Guangzhou China gdpu.edu.cn

**Keywords:** candidate biomarkers, IgAN, immunoglobulin A nephropathy, machine learning, vascular calcification-related pathways

## Abstract

**Background:**

Immunoglobulin A nephropathy (IgAN) is one of the most prevalent primary glomerular diseases globally. This study is aimed at identifying candidate biomarkers associated with vascular calcification‐related signaling pathways in IgAN and at investigating their potential biological associations.

**Methods:**

This was an observational bioinformatics study based on public transcriptomic datasets. Vascular calcification‐related genes (VCRGs) were defined based on selected KEGG signaling pathways. Data were obtained from public databases. Candidate biomarkers were identified via differential analysis, machine learning, and cross‐compartment external expression assessment. Enrichment analysis, immune infiltration, regulatory networks, computational drug prediction, and computational N6‐methyladenosine (m6A) site prediction were used to explore the potential biological context of the candidate biomarkers.

**Results:**

Differential analysis identified 348 differentially expressed genes (DEGs). By intersecting these DEGs with VCRGs, 30 candidate genes were obtained. *JUN*, *FOS*, and *COL1A2* were finally identified as candidate biomarkers, and these genes were mainly associated with the “Nagashima EGF signaling” and “Nagashima NRG1 signaling.” Six differential immune cell types (e.g., M2 macrophages) differed significantly between the IgAN and control groups. Spearman analysis showed that COL1A2 was negatively correlated with resting natural killer (NK) cells (cor = −0.57, *p* < 0.05); and FOS was positively correlated with resting NK cells (cor = 0.72, *p* < 0.01). BRD4, MAZ, and CTCF were computationally predicted as shared candidate transcription factors (TFs). A regulatory association involving hsa‐miR‐139‐5p and FOS was also predicted. Troglitazone was predicted to target JUN. High‐confidence m6A modification sites were computationally predicted in all three candidate biomarkers. These findings were considered exploratory and hypothesis generating.

**Conclusion:**

*JUN*, *FOS*, and *COL1A2* were identified as IgAN‐associated candidate biomarkers linked to vascular calcification‐related signaling pathway genes, providing exploratory biological insights into the potential molecular links between IgAN and calcification‐related signaling.

## 1. Introduction

Immunoglobulin A nephropathy (IgAN) is a kidney disease characterized by “IgA‐IgG deposits in the mesangial capillaries” of the glomeruli. It is the most common primary glomerular disease and remains a leading cause of chronic kidney disease (CKD) and end‐stage renal disease (ESRD) [1]. The current diagnostic gold standard is still renal biopsy [2]. Although corticosteroids can reduce proteinuria, their long‐term benefits are questionable, and their use is associated with serious adverse effects, particularly infections [3]. Therefore, molecular‐level research into IgAN is needed to develop novel therapeutic strategies targeting its pathogenesis.

Vascular calcification is classified into three types based on location: intimal, medial, and valvular. Vascular calcification is associated with multiple cell signaling pathways, including Wnt, mitogen‐activated protein kinase (MAPK), phosphatidylinositol‐3‐kinase (PI3K)/Akt, cyclic nucleotide‐dependent protein kinase, protein kinase C (PKC), calcium/calmodulin‐dependent kinase II (CaMKII), adenosine monophosphate‐activated protein kinase (AMPK)/mammalian target of rapamycin (mTOR), Ras homolog (Rho) GTPases, apoptosis, Notch, and cytokine signaling pathways [4]. Vascular calcification is a common and significant cardiovascular risk factor in patients with CKD [5]. To date, there are no clinically feasible therapies approved for treating and preventing vascular calcification, or validated early biomarkers. This is partly due to the asymptomatic progression of calcification and the lack of high‐resolution imaging techniques to identify early calcification before it reaches a point of no return [6]. It is worth noting that the molecular mechanism of vascular calcification in IgAN patients has not been clarified, and effective early biomarkers have been lacking. Patients with IgAN are at a significantly increased risk of cardiovascular disease, for which vascular calcification is a key pathological hallmark. Growing evidence suggests that vascular calcification and IgAN progression share several underlying pathophysiological mechanisms. For instance, both conditions are associated with a chronic inflammatory state [7, 8], elevated oxidative stress [9, 10], activation of the renin–angiotensin system [11, 12], and dysregulation of shared cellular signaling pathways, such as the Wnt/*β*‐catenin and BMP/Smad pathways [13, 14]. This common mechanistic ground implies that genes involved in vascular calcification that are dysregulated in IgAN may not merely be markers of cardiovascular comorbidity but could play direct roles in renal injury and fibrosis inherent to IgAN itself. Consequently, a systematic investigation of the expression patterns and functions of vascular calcification‐related genes (VCRGs) in IgAN offers an opportunity to understand disease progression from a novel perspective and to identify candidate biomarkers with potential prognostic value for both renal and cardiovascular outcomes.

To address this clinical challenge, machine learning (ML) approaches have provided new solutions for identifying reliable candidate biomarkers from complex omics data. ML, as an advanced data analytics tool, has demonstrated significant potential in recent research on IgAN and vascular calcification. Through deep mining of extensive clinical and omics data, ML algorithms can extract complex feature patterns and establish precise risk prediction models. This enables critical contributions to early screening, prognostic evaluation, and treatment decision‐making. For example, by integrating patient immune markers, inflammatory cytokine levels, and metabolic parameters, ML models can effectively identify high‐risk IgAN patients and predict their vascular calcification progression trends, providing data‐driven support for clinical management. Furthermore, integrated analysis of multiomics data helps uncover underlying molecular mechanisms, laying the groundwork for personalized treatment approaches.

The core hypothesis of this study is that key genes associated with vascular calcification‐related signaling pathways can be identified as novel candidate biomarkers in IgAN patients using transcriptomic data and ML. This study utilizes publicly available transcriptomic data from IgAN patients, combined with genes related to the hot research topic of vascular calcification. Utilizing differential expression analysis, ML, and expression level validation, it aims to identify candidate biomarkers associated with vascular calcification in IgAN. It further seeks to understand the biological functions these candidate biomarkers participate in, their association with the immune microenvironment, the molecular regulatory networks they are involved in, and potential therapeutic drug predictions. This approach could provide new insights for IgAN treatment.

## 2. Materials and Methods

### 2.1. Data Acquisition

IgAN datasets were obtained from the Gene Expression Omnibus (GEO) database (https://www.ncbi.nlm.nih.gov/geo/). The GSE93798 (GPL22945) dataset, comprising glomerular samples from 20 IgAN patients (IgAN group) and 22 control subjects (control group), was used as the training dataset. The GSE175759 (GPL16791) dataset, comprising tubulointerstitial samples from 46 IgAN patients (IgAN group) and 22 control subjects (control group; other samples within this dataset were excluded), was profiled on an alternate platform and applied for cross‐compartment external expression evaluation instead of rigorous independent replication. Additionally, 709 VCRGs were obtained from the Kyoto Encyclopedia of Genes and Genomes (KEGG) database. These genes were identified based on the following pathways: hsa04020, hsa04270, hsa04978, hsa04933, hsa04350, and hsa05417 (Table S1). All the above datasets are publicly available and can be freely accessed via the official websites of GEO and KEGG databases.

### 2.2. Candidate Genes Acquisition

The limma package (v 3.56.2) [15] was utilized to identify differentially expressed genes (DEGs) between the IgAN and control groups in the GSE93798 dataset with false discovery rate (FDR) < 0.05 and |log2 FC| > 1. Using ggplot2 (v 3.4.1) (https://cran.r-project.org/web/packages/ggplot2/index.html), a volcano plot was constructed to visualize DEGs, with labels for the top 10 upregulated and downregulated genes. The pheatmap package (v 2.14.0) (https://cran.r-project.org/web/packages/pheatmap/) was applied to create a heatmap showcasing expression patterns of the top 10 genes from both categories.

The ggvenn package (v 0.1.10) (https://cran.r-project.org/web/packages/ggvenn/) was used to integrate the DEGs and VCRGs. The obtained genes were designated as candidate genes. Enrichment analysis of candidate genes was conducted using the clusterProfiler package (v 4.15.0.3) [16], including Gene Ontology (GO) (Benjamini–Hochberg, FDR < 0.05) and KEGG enrichments (Benjamini–Hochberg, FDR < 0.05). The top five terms for each category and the top 10 KEGG pathways were displayed based on the number of enriched genes.

### 2.3. Candidate Biomarkers Acquisition

A protein–protein interaction (PPI) network was established based on candidate genes using the Search Tool for the Retrieval of Interacting Genes (STRING) database (interaction score ≥ 0.4, a minimum required interaction score of 0.4 was used, corresponding to the medium‐confidence threshold in STRING, to balance interaction reliability with sufficient network coverage for downstream topology analysis). The result was visualized using Cytoscape (v 3.9.1) [17]. Next, network topological analysis (maximal clique centrality [MCC], maximum neighborhood component [MNC], edge percolated component [EPC], degree, and closeness) was performed on the candidate genes using the cytoHubba plugin in Cytoscape (v 3.9.1). The top 10 genes from the five algorithms were integrated using the ggvenn package (v 0.1.10), and the resulting genes were termed key genes. Meanwhile, an interaction network among the key genes was constructed. A network‐guided sequential feature‐selection strategy was adopted, in which network‐topology analysis was first used to prioritize candidate genes and reduce the feature space, followed by complementary ML algorithms for further feature refinement. This strategy was intended to limit model complexity given the relatively small sample size.

The nine key genes identified by cytoHubba were used as the common input features for subsequent ML analyses. The least absolute shrinkage and selection operator (LASSO) regression analysis was performed on these nine key genes using the glmnet package (v 4.1‐8) [18] with fivefold cross‐validation, and genes retained at lambda min were referred to as Feature Genes 1. The support vector machine‐recursive feature elimination (SVM‐RFE) analysis was performed on these nine key genes using the caret package (v 6.0‐94) (https://cran.r-project.org/web/packages/caret/index.html) with fivefold cross‐validation. During recursive feature elimination, feature subsets containing one to nine genes were sequentially evaluated. Model performance was assessed using cross‐validated classification accuracy, with the classification error calculated as 1 = accuracy. The feature subset yielding the highest cross‐validated accuracy was selected as Feature Genes 2. Random forest (RF) analysis was also performed using the same nine key genes as input with the randomForest package (v 4.7.1.2) (https://cran.r-project.org/package=randomForest). Candidate ntree values ranging from 1 to 500 were evaluated at increments of 5 using fivefold cross‐validation. The optimal ntree value was selected based on the lowest mean cross‐validated classification error rate, which was achieved at ntree = 6. Based on the corresponding variable importance ranking, the top three genes were designated as Feature Genes 3. Shared feature genes were identified by the intersection of Feature Genes 1, 2, and 3 using the ggvenn package (v 0.1.10). Finally, Wilcoxon test (unadjusted *p* < 0.05) was applied to compare the expression levels of these shared feature genes in the GSE93798 and GSE175759 datasets between the IgAN and control groups. Genes demonstrating significant expression differences with consistent trends across both datasets were selected and designated as candidate biomarkers. Because the two datasets differed in renal compartment and platform, consistency of expression direction was interpreted as cross‐compartment support rather than strict independent replication.

### 2.4. Gene Set Enrichment Analysis (GSEA)

GSEA was performed using the clusterProfiler package (v 4.15.0.3) with the “c2.all.v2023.2.Hs.symbols.gmt” gene set from MSigDB and the fgsea algorithm. The exponent was set to 1, and gene‐set sizes were restricted to 10–500 genes (minGSSize = 10, maxGSSize = 500). The numerical boundary parameter eps was set to 1 × 10^−10^. Enrichment significance was estimated using the adaptive multilevel splitting Monte Carlo procedure implemented in fgsea, with nPermSimple = 1000 for initial *p* value estimation and a random seed of 123 for reproducibility. Multiple‐testing correction was performed using the Benjamini–Hochberg method. Gene sets with |*N* *E* *S*| > 1, nominal p < 0.05, and *q* value < 0.25 were considered significant. The correlation coefficients between each candidate biomarker and all genes in the GSE93798 dataset were computed using the psych package (v 2.4.3) (https://cran.r-project.org/package=psych). The five gene sets with the highest and lowest NES values were displayed for each candidate biomarker.

### 2.5. Immune Microenvironment Analysis

The CIBERSORT algorithm was applied to assess the proportions of 22 immune cell types in the GSE93798 dataset. Next, the Wilcoxon test was performed to identify differential scores of immune cell types between the IgAN and control groups (*p* < 0.05). Multiple‐testing correction was performed using the Benjamini–Hochberg method, with adjusted *p* < 0.05 considered statistically significant. Finally, Spearman correlation analysis was conducted on all samples of the GSE93798 dataset to examine the relationships between candidate biomarkers and differential immune cell types, as well as between the differential immune cell types themselves, using the psych package (|*c* *o* *r* *r* *e* *l* *a* *t* *i* *o* *n* *c* *o* *e* *f* *f* *i* *c* *i* *e* *n* *t* | > 0.30, *p* < 0.05) (v 2.4.3). Bootstrap analysis (1000 iterations) was performed to calculate cell proportion means and 95% percentile CIs; zero‐inflation and estimate robustness were evaluated using the zero proportion and coefficient of variation (CV), respectively.

### 2.6. Regulation Network Analysis and Computational Drug Prediction

Transcription factors (TFs) associated with candidate biomarkers were predicted through the CHIPBase and hTFtarget databases. The results from the two databases were integrated to identify the key TFs. The miRDB and ENCORI databases were used to predict microRNAs (miRNAs) associated with the candidate biomarkers. The results from the two databases were integrated to identify the key miRNAs. Finally, the key miRNA‐mRNA and key TF‐mRNA networks were visualized using Cytoscape (v 3.9.1).

Additionally, to predict drugs associated with candidate biomarkers, drugs were identified using the DSigDB. All drug–gene associations were regarded as computationally predicted candidates and were not interpreted as evidence of clinical efficacy. The drug‐candidate biomarker network was then visualized using Cytoscape (v 3.9.1) (only drugs associated with all candidate biomarkers were displayed).

### 2.7. Computational Molecular Docking

Based on the results of drug prediction, drugs related to the disease in the present study were selected for molecular docking. First, drugs were searched in the PubChem database (https://pubchem.ncbi.nlm.nih.gov/) to obtain their three‐dimensional (3D) structures. Second, candidate biomarkers were retrieved from the Protein Data Bank (PDB) (https://www.rcsb.org/) to acquire the receptor structures with the highest resolution. Subsequently, molecular docking was performed using the CB‐DOCK2 webserver. A docking score below −5 kcal/mol was interpreted as indicating potentially favorable predicted binding affinity. Molecular docking was used only to assess theoretical ligand–protein interactions and did not establish biological activity or therapeutic efficacy.

### 2.8. Computational Prediction of N6‐Methyladenosine (m6A) Sites

First, the corresponding nucleic acid sequences of the candidate biomarkers were retrieved from the NCBI database. The candidate biomarker sequences were then input into the SRAMP database (http://www.cuilab.cn/m6asiteapp/old) to predict their m6A modification sites.

### 2.9. Statistical Evaluation

R (v 4.3.1) was used to conduct the bioinformatics analysis. Wilcoxon rank‐sum test was used for comparison between two groups. Since only a small number of prespecified key genes were tested, no multiple testing correction (FDR/Bonferroni) was applied. Multiple‐testing correction was applied to DEG, enrichment, GSEA, and immune infiltration analyses. TF, miRNA, and drug predictions were database‐based and did not involve *p* value testing. All statistical tests were two‐sided, and *p* < 0.05 was considered statistically significant. This experiment does not require ethical approval.

## 3. Results

### 3.1. Acquisition of 30 Candidate Genes

In the GSE93798 dataset, 348 DEGs were identified, with 107 genes upregulated and 241 genes downregulated in the IgAN group (Figure 1A,B). By integrating DEGs and VCRGs, 30 candidate genes were identified (Figure 1C). Candidate genes were significantly associated with 167 GO terms and 45 KEGG pathways. For instance, GO terms included “oxidoreductase activity, acting on NAD(P)H, oxygen as acceptor,” “positive regulation of cytokine production,” and “NADPH oxidase complex” (Figure 1D, Table S2). The KEGG pathways primarily included “lipid and atherosclerosis” and “MAPK signaling pathway” (Figure 1E, Table S3).

**Figure 1:**
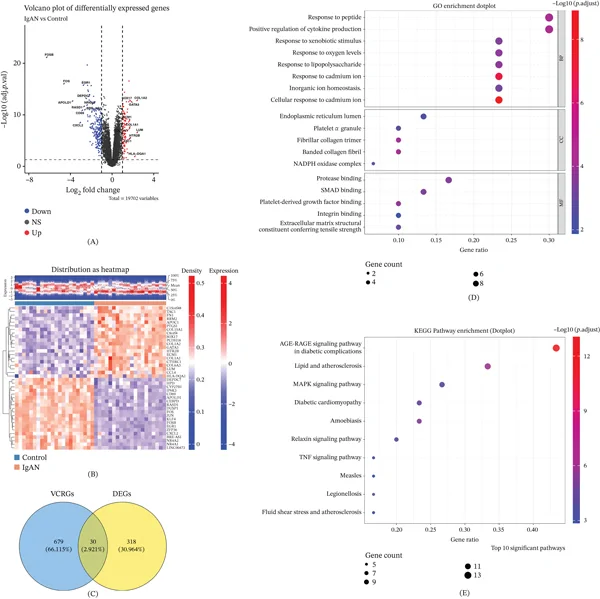
Determination of candidate genes. (A) Volcano plot of differentially expressed genes. Red: upregulated genes; blue: downregulated genes. The top 10 upregulated and downregulated genes were shown. (B) Heatmap of differential gene expression. Orange: IgAN group; purple: control group; red: high expression; blue: low expression. (C) Venn diagram of candidate genes. Thirty candidate genes were obtained. (D) GO enrichment analysis of candidate genes. Dot size represents the number of genes associated with each GO term, and dot color represents statistical significance. (E) KEGG enrichment analysis of candidate genes. The size of the dots represents the number of genes in the pathway, and the color of the dots represents significance.

### 3.2. Identification of JUN, FOS, and COL1A2 as Candidate Biomarkers

In the PPI network, 30 interacting proteins were identified, such as *JUN* and *FOS* (Figure 2A). A total of nine key genes (*JUN*, *FN1*, *FOS*, *COL1A2*, *IL1B*, *COL1A1*, *SERPINE1*, *MMP2*, and *AGT*) were identified using five types of network topological analysis (Figure 2B). The interaction network of key genes indicated that both *JUN* and *FOS* interacted more strongly with *IL1B* (Figure 2C).

**Figure 2:**
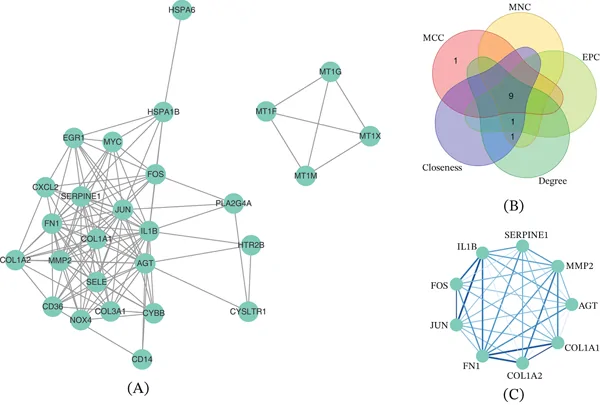
Acquisition and analysis of key genes. (A) PPI network of the candidate genes. Nodes represented genes, whereas edges represented interactions between genes. (B) Identification of key genes. Nine key genes were obtained. (C) The interaction network of key genes. Edge thickness and color intensity represent the combined interaction score, with thicker and darker edges indicating higher scores.

A total of six Feature Genes 1 were obtained using the LASSO regression analysis (lambda.min = 0.0004332147) (Figure 3A). A total of three Feature Genes 2 were identified using the SVM‐RFE algorithm (Figure 3B). Meanwhile, three Feature Genes 3 were identified using the RF algorithm (ntree = 6) (Figure 3C). By integrating Feature Genes 1, 2, and 3, three key genes were identified (Figure 3D). Further expression analysis of candidate biomarkers in the GSE93798 and GSE175759 datasets revealed that *JUN* and *FOS* were significantly downregulated in the IgAN group, whereas *COL1A2* was significantly upregulated in the IgAN group. Therefore, *JUN*, *FOS*, and *COL1A2* were classified as candidate biomarkers (*p* < 0.05) (Figure 3E).

**Figure 3:**
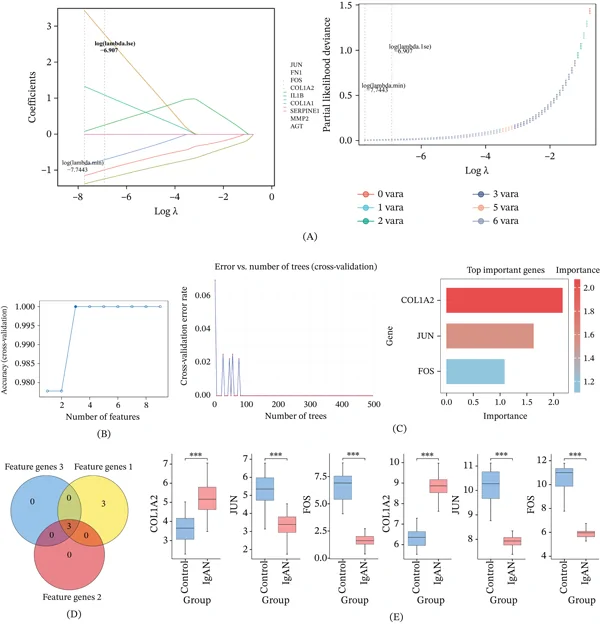
Identification of biomarkers. (A) Least absolute shrinkage and selection operator (LASSO) analysis. Left: LASSO coefficient profiles of candidate genes. Right: fivefold cross‐validation for selection of the optimal *λ* value. (B) SVM‐RFE analysis results. *x*‐axis: number of feature genes; *y*‐axis: model prediction accuracy. (C) Left: average classification error rates at different ntree values. Right: variable importance ranking of candidate genes. (D) Venn diagram of key genes. Three key genes were obtained. (E) Differential expression levels of candidate biomarkers in GSE93798 and GSE175759. ∗∗∗: unadjusted *p* < 0.001. Blue: control group; red: IgAN group.

### 3.3. Multiple Signaling Pathways in IgAN

The GSEA revealed that *JUN* was enriched in 1480 pathways, including “Nagashima EGF signaling”; *FOS* was enriched in 1528 pathways, including “Nagashima NRG1 signaling”; and *COL1A2* was enriched in 1722 pathways, including “Nagashima EGF signaling,” with thresholds set at |*N* *E* *S*| > 1, nominal *p* < 0.05, and *q* value < 0.25. The pathways commonly enriched by the three candidate biomarkers included “Nagashima EGF signaling” and “Nagashima NRG1 signaling,” among others (Figure 4A–C; Tables S4, S5, and S6). The enrichment findings represent statistical associations and do not establish direct pathway activation or causal involvement in IgAN.

**Figure 4:**
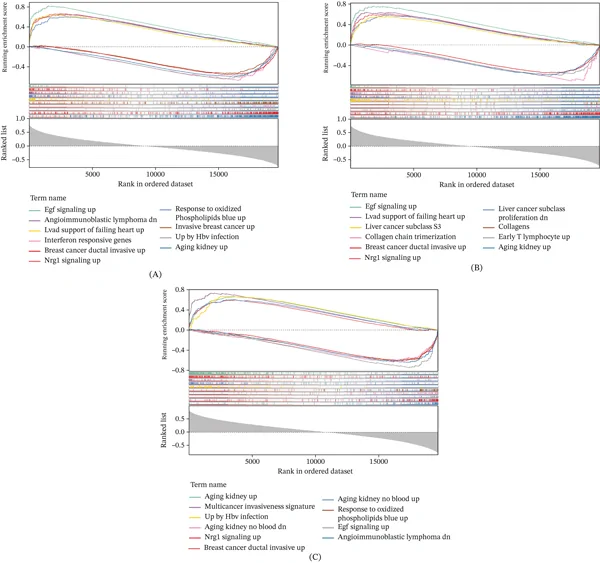
GSEA of biomarkers. (A–C) Gene set enrichment analysis (GSEA) of JUN, FOS, and COL1A2. Each line represented a pathway, and the peak of each line was the enrichment score of that pathway. The genes before the peak were the core genes in the gene set of that pathway. The peak in the upper left corner represented that the core genes were mainly positively correlated with biomarkers, whereas the peak in the lower right corner represented that the core genes were mainly negatively correlated with biomarkers.

### 3.4. Association of Candidate Biomarkers With the Immune Microenvironment

The stacked plot demonstrated the proportions of 21 immune cell types in IgAN and control groups (one immune cell type was not detected) (Figure 5A). The scores of six immune cell types showed significant differences, including M2 macrophages (*p* < 0.05) (Figure 5B). After Benjamini–Hochberg correction, only resting NK cells showed a significant difference between the IgAN and control groups (adjusted *p* < 0.05), with a lower estimated proportion in the IgAN group (Figure S1). Spearman correlation analysis revealed that gamma delta T cells showed the strongest correlation with eosinophils (cor = 1, *p* < 0.05) (Figure 5C). Data distribution and robustness analyses revealed that only one sample had nonzero values for both gamma delta T cells and eosinophils. Bootstrap resampling analysis with 1000 iterations showed that the 95% confidence interval of the correlation coefficient was [1], suggesting that the perfect correlation (cor = 1) resulted from an extremely sparse, zero‐inflated data structure and represented a methodological artifact rather than a genuine biological association. This finding did not affect the main conclusions of the study regarding gene‐immune cell correlations. Subsequent research focused on the results of the robustness analysis. In addition, Spearman correlation analysis revealed that *COL1A2* showed the strongest correlation with resting natural killer (NK) cells (cor = −0.57, *p* < 0.05), and *FOS* with resting NK cells (cor = 0.72, *p* < 0.01) (Figure 5D). These findings suggest that the identified candidate biomarkers may be associated with alterations in the inferred immune landscape of IgAN, although functional studies are required to determine the underlying biological relationships. Analysis of the CV indicated that the reliability of immune cell proportion differences was robust overall (Figure 5E). Specifically, a CV < 0.1 indicates excellent robustness, 0.1 < CV < 0.3 indicates good robustness, and CV ≥ 0.3 indicates poor robustness.

Figure 5Immune microenvironment analysis of biomarkers. (A) Stacked plot of immune cell infiltration ratio in IgAN and control groups. Different colors represented different immune cells. (B) Comparison of estimated immune cell proportions between the IgAN and control groups. (C) Correlations among differential immune cells. The depth of color represented the magnitude of the correlation coefficient. (D) Correlations between biomarkers and differential immune cells. The depth of color represented the magnitude of the correlation coefficient. ∗: *p* < 0.05; ∗∗: *p* < 0.01; ∗∗∗: *p* < 0.001. (E) The coefficient of variation (CV) confirmed the robustness of immune cell proportion differences, with values classified as excellent (< 0.1), good (0.1–0.3), or poor (≥ 0.3).

**Figure:**
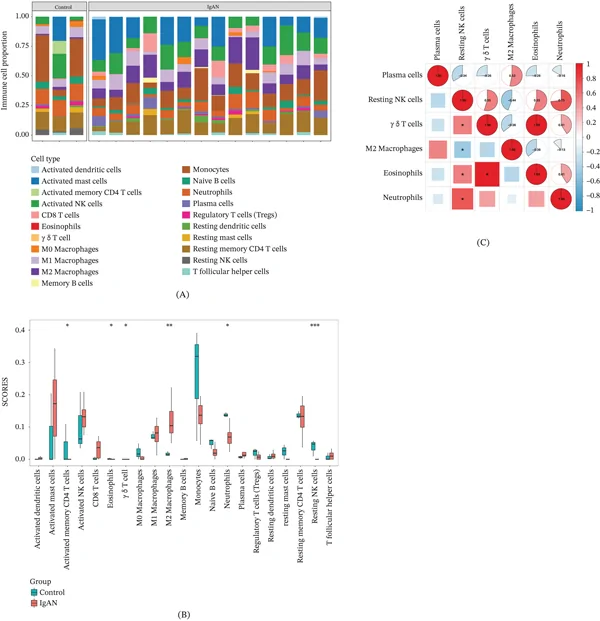

**Figure:**
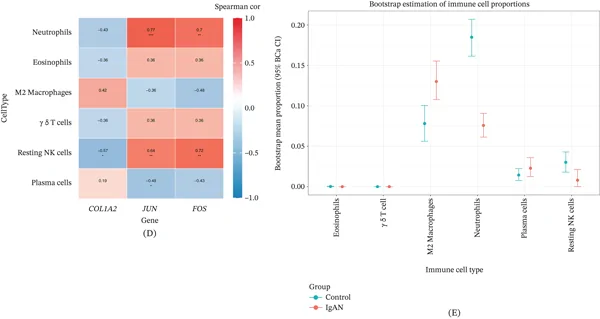

### 3.5. Exploring the Molecular Regulatory Mechanisms of Candidate Biomarkers

A total of 76 key TFs were shared among *JUN*, 40 key TFs were associated with *FOS*, and four key TFs were associated with *COL1A2*. A total of three key TFs were associated with *JUN*, *FOS*, and *COL1A2*, including *BRD4*, *MAZ*, and *CTCF* (Figure 6A). A total of 16 key miRNAs were associated with *JUN*, 17 key miRNAs were associated with *FOS*, and 34 key miRNAs were associated with *COL1A2*. A total of one key miRNA, hsa‐miR‐139‐5p, was associated with *FOS* and *JUN*, whereas two key miRNAs, hsa‐miR‐524‐5p and hsa‐miR‐520d‐5p, were associated with *COL1A2* and *JUN*. The identified regulatory networks included hsa‐miR‐139‐5p‐*FOS* and hsa‐miR‐139‐5p‐*JUN* (Figure 6B). These potential regulatory mechanisms may have implications for the development of IgAN.

**Figure 6:**
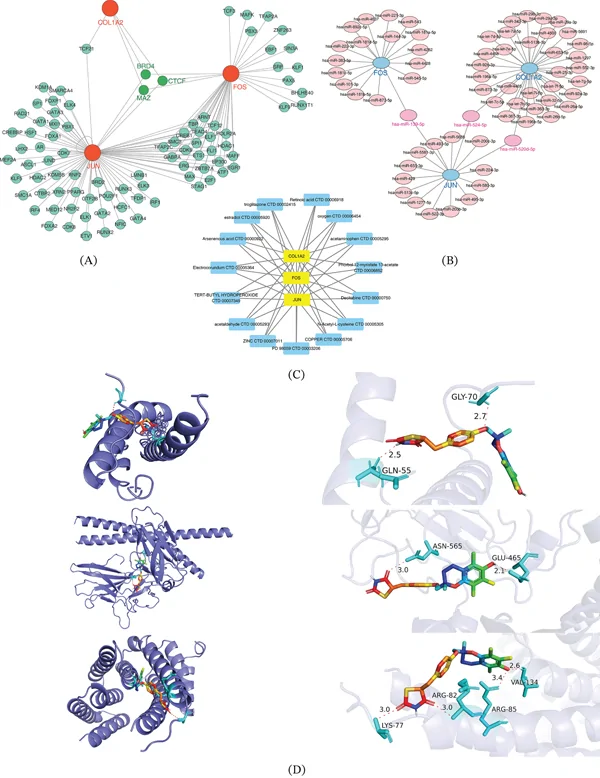
Computational prediction of regulatory associations and candidate compounds. (A) Predicted transcription factor (TF)–mRNA regulatory network of the biomarkers. Orange: biomarker; green: TF. The connections represent the existence of regulatory relationships. (B) Predicted microRNA (miRNA)–mRNA regulatory network of the biomarkers. Blue: biomarker; pink: miRNA. Edges represent computationally predicted associations. (C) Computationally predicted compound–biomarker association network. Yellow: biomarker; blue: drug. A higher degree is represented by a larger node size. The connections represent the existence of regulatory relationships. (D) Results of molecular docking analysis of biomarkers (COL1A2, FOS, and JUN) with the drug troglitazone.

Additionally, drugs such as electrocorundum, acetaldehyde, and troglitazone were all associated with the candidate biomarkers (Figure 6C). These compounds were identified as computational candidates potentially associated with the candidate biomarkers and should not be interpreted as clinically applicable treatments for IgAN. For example, Protein kinase A inhibitors may suppress the activation of oxidative stress‐related signaling pathways, reduce the generation of reactive oxygen species, alleviate oxidative stress‐induced damage to renal cells, and may help preserve renal function. Oxidative stress is a recognized key driver in the progression of IgAN, contributing to renal damage through multiple mechanisms including lipid peroxidation and inflammation [19].

Since troglitazone was more relevant to the disease in the present study, this drug was selected for molecular docking. Moreover, troglitazone has demonstrated favorable therapeutic effects in diabetic nephropathy [20] and renal fibrotic lesions [21]. Molecular docking results indicated that COL1A2, FOS, and JUN all exhibited favorable binding affinity with troglitazone, with binding energies of −8.7, −7.6, and −8.1 kcal/mol, respectively. These results suggested that troglitazone may have important research significance in IgAN and may exert its effects by binding to FOS, JUN, and COL1A2 (Figure 6D). Notably, troglitazone has been withdrawn from clinical use because of serious hepatotoxicity; therefore, it was considered only as a computationally predicted tool compound in the present study and not as a therapeutic recommendation.

### 3.6. Computational Prediction of Putative m6A Sites in the Candidate Biomarkers

The m6A methylation site results indicated that all three candidate biomarkers possessed highly confident methylation sites (Figure 7A–C). These findings provide hypotheses for future experimental investigation but do not confirm that the predicted sites are functionally modified or involved in IgAN.

**Figure 7:**
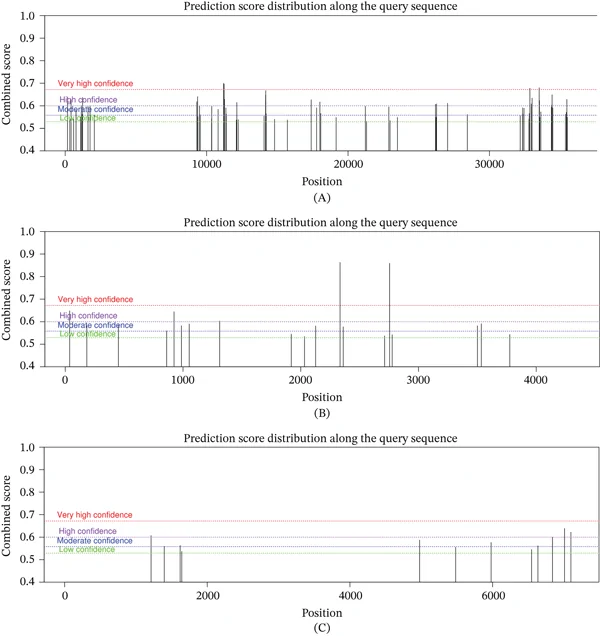
Computational prediction of putative m6A sites in the biomarkers. (A–C) Predicted putative m6A sites in JUN, FOS, and COL1A2 transcripts. Green indicates low confidence, blue indicates moderate confidence, purple indicates high confidence, and red indicates very high confidence.

## 4. Discussion

IgAN, one of the most prevalent primary glomerular diseases worldwide, is frequently associated with vascular calcification in patients. This study employs bioinformatics approaches, integrating transcriptomic data with ML techniques, to identify three IgAN‐associated candidate biomarkers: *JUN*, *FOS*, and *COL1A2*. This study performed multiple analyses, including enrichment analysis, immune microenvironment profiling, molecular regulatory network construction, drug prediction, and m6A methylation site prediction. These analyses provide new insights into potential therapeutic avenues for IgAN. In addition, the cross‐tissue validation approach used in this study deserves special discussion. Although glomerular injury represents a characteristic feature of early IgAN, progression to CKD involves tubulointerstitial fibrosis and vascular pathological changes. Our study found that *JUN*, *FOS*, and *COL1A2* exhibited consistent expression patterns in both glomerular and tubulointerstitial compartments. A limitation of this study is the use of datasets from different renal compartments (glomeruli vs. tubulointerstitium) for training and validation. Glomerular and tubulointerstitial tissues differ in their cellular composition and tissue‐specific transcriptional profiles, which may influence gene expression levels, effect sizes, and statistical significance. Therefore, the concordant expression trends observed across the two datasets should be interpreted as cross‐compartment supportive evidence rather than strict independent replication in a homogeneous external cohort. Nevertheless, the consistent dysregulation of the identified candidate biomarkers across both renal compartments provides additional support for their potential association with IgAN. Further validation in independent cohorts with matched tissue compartments and comparable transcriptomic platforms is warranted.

Although Zhou et al. [22] previously reported that *JUN* and *FOS* could serve as genes associated with distinguishing patients with IgAN from controls, the added value of the present study did not lie in claiming the first identification of *JUN* and *FOS*, but rather in placing these two genes within an analytical framework focused on vascular calcification‐related pathways. Specifically, we integrated DEGs in IgAN with a predefined set of VCRGs and subsequently performed comprehensive screening using network topology analysis, LASSO, SVM‐RFE, and RF algorithms. This strategy further highlighted *COL1A2* as an additional candidate biomarker with potential relevance. Moreover, we extended the analysis by evaluating associations with immune cell infiltration, constructing TF–mRNA and miRNA–mRNA regulatory networks, and conducting exploratory computational drug prediction, molecular docking, and m6A site prediction. Collectively, these analyses provided a broader regulatory and functional context for the identified biomarkers and generated new hypotheses for subsequent experimental validation.

JUN and FOS were significantly downregulated in IgAN in the present study, consistent with the findings of Zhou et al. [22], who previously identified these genes as potential markers distinguishing patients with IgAN from healthy controls. JUN and FOS are components of the AP‐1 transcriptional regulatory system and have been implicated in inflammatory, cellular injury, and stress‐response processes in the kidney [23–25]. However, their specific functional roles in IgAN remain unclear. Rather than representing the initial discovery of JUN and FOS, the present study extends previous findings by placing these genes within a vascular calcification‐related transcriptomic framework and further characterizing their associated pathways, immune signatures, and predicted regulatory relationships.

COL1A2 was upregulated in both the glomerular and tubulointerstitial IgAN datasets and represents an additional candidate biomarker highlighted by the present analysis. Previous studies have associated COL1A2 with extracellular matrix deposition, tissue remodeling, and renal fibrosis in CKD [26–29]. Therefore, its increased expression in IgAN may be associated with fibrotic remodeling across renal compartments. However, because fibrosis severity and COL1A2 function were not directly assessed, this interpretation remains exploratory and requires tissue‐based and functional validation.

GSEA results reveal that *JUN*, *FOS*, and *COL1A2* were commonly associated with the “Nagashima EGF signaling” and “Nagashima NRG1 signaling” gene sets. These findings suggest that the three candidate biomarkers may be involved in EGF‐ and NRG1‐related signaling processes in IgAN rather than direct experimental evidence of pathway activation. EGF‐ and NRG1‐related signaling has been implicated in renal injury, repair, inflammation, and fibrotic remodeling. Following AKI, activation of the EGF/EGFR axis may promote tubular epithelial cell proliferation and renal recovery while attenuating inflammatory responses [30]. However, persistent or dysregulated EGF‐related signaling during CKD progression may be associated with maladaptive remodeling. In IgAN, tubulointerstitial EGF expression has been correlated with the severity of tubular atrophy and interstitial fibrosis [31], and EGF‐related signaling has also been linked to extracellular matrix production and fibroblast‐to‐myofibroblast transition [32]. NRG1/ErbB signaling contributes to renal development and has been associated with cell survival, inflammatory responses, and tissue repair [33]. In experimental diabetic nephropathy, NRG1 has shown potential protective effects on podocytes through antiapoptotic and anti‐inflammatory processes [34]. Collectively, the enrichment of the EGF‐ and NRG1‐related gene sets suggests that these signaling processes may be associated with fibrotic remodeling and renal injury responses in IgAN. However, these findings are based on statistical enrichment and do not establish pathway activation, causal involvement, or a direct mechanistic connection with vascular calcification. Further experimental studies are required to clarify their biological relevance in IgAN.

Immunological infiltration analysis revealed significant differences in six immune cell types between IgAN and control groups, including NK cells, M2 macrophages, eosinophils, and others. Within the tumor microenvironment of renal cancers, M2‐like macrophages have been associated with tumor‐promoting activities, including angiogenesis, extracellular matrix remodeling, and tumor cell survival [35]. Conversely, in nonneoplastic contexts, M2‐like macrophages may contribute to immune regulation and tissue repair by limiting excessive inflammatory responses [36]. These findings suggest that alterations in the immune cell landscape may be associated with IgAN, which is characterized by immune complex deposition and chronic renal inflammation. It should be noted that CIBERSORT estimates relative immune cell proportions from bulk transcriptomic data. Therefore, the observed differences and associations represent computationally inferred immune signatures rather than experimentally demonstrated cell–cell interactions or regulatory mechanisms.

Additionally, in computational drug prediction results, compounds associated with all candidate biomarkers (JUN, FOS, and COL1A2) identified via DSigDB include electrocorundum (a nanomaterial) [37], acetaldehyde (a metabolite) [38], and troglitazone. It should be noted that these are bioinformatically screened candidate compounds, not clinically recommended drugs, but their basic research significance is worth exploring: Electrocorundum, as a nanomaterial, may mediate vascular calcification‐related pathways via targeted drug delivery; acetaldehyde, although a toxic metabolite, may act as a signaling molecule regulating oxidative stress at low concentrations [39]; troglitazone, though withdrawn due to hepatotoxicity, has been shown to have antifibrotic effects in diabetic nephropathy [20] and renal fibrosis [21] in previous studies, serving as a tool drug for mechanism research. Experimental pharmacological studies would be required to determine whether any of the predicted compounds have biological relevance in IgAN.

*CTCF* is a conserved chromatin organizer involved in the transcriptional regulation of renal genes, including genes associated with the renin phenotype [40]. In the present study, CTCF was computationally predicted as a shared candidate TF associated with JUN, FOS, and COL1A2. However, this predicted association does not confirm direct transcriptional regulation or establish its functional relevance in IgAN. m6A RNA modification has been implicated in several kidney diseases and may influence inflammation, apoptosis, fibrosis, and podocyte injury [41, 42]. For example, METTL3‐mediated m6A regulation has been associated with obstructive renal fibrosis through the SPRY1/ERK/NF‐*κ*B pathway [43] and with podocyte injury in diabetic nephropathy through modification of TIMP2 mRNA [44]. These previous findings provide a general biological context for investigating m6A modification in kidney disease. Nevertheless, the present study only used SRAMP to computationally predict putative m6A sites in JUN, FOS, and COL1A2. Therefore, these results do not confirm actual m6A modification, functional regulation, or involvement of the predicted sites in IgAN and should be regarded as hypothesis‐generating.

Through bioinformatics analysis, this study identified three VCRG‐associated candidate biomarkers: *JUN*, *FOS*, and *COL1A2*. We further characterized their biological functions, associations with the immune microenvironment, molecular regulatory networks, potential targeted drugs, and m6A methylation modification patterns. These findings provide novel insights into the mechanisms and therapeutic strategies of IgAN. Nevertheless, several limitations should be acknowledged. First, all results were obtained from public transcriptome data; thus, in vitro functional experiments (e.g., cell proliferation, apoptosis, and fibrosis‐related assays in renal mesangial or tubular cells) and in vivo animal models (e.g., IgAN mouse models) are required to verify the expression and biological roles of *JUN*, *FOS*, and *COL1A2*. Second, the available public datasets lacked vascular calcification phenotypes, and no suitable independent public IgAN cohort with well‐characterized vascular calcification information was available for further validation. Therefore, prospective clinical cohorts with larger sample sizes and vascular calcification phenotyping are needed to validate these genes as candidate biomarkers and clarify their potential relevance to vascular calcification in IgAN. Third, LASSO, SVM‐RFE, and RF were performed on the nine key genes preselected by cytoHubba rather than all 30 candidate genes. Thus, the ML analyses were not fully independent of the preceding network‐topology filtering and may have introduced feature‐selection bias. Future studies should validate these findings using feature selection directly from the complete candidate‐gene set. Finally, the immune infiltration, GSEA, regulatory network, drug prediction, molecular docking, and m6A site analyses were entirely computational or association‐based. Therefore, these findings should be regarded as exploratory and hypothesis generating, and require validation through cellular, molecular, and pharmacological experiments.

## 5. Conclusions

This study analyzed transcriptomic data (GSE93798 and GSE175759) and VCRGs via bioinformatics approaches, ultimately identifying JUN, FOS, and COL1A2 as candidate biomarkers associated with vascular calcification‐related signaling pathway genes in IgAN. Exploratory computational analyses further indicated that these candidate biomarkers were enriched in EGF/NRG1 signaling pathways and related to differential immune cells (e.g., M2 macrophages). TF/miRNA regulatory networks and high‐confidence m6A sites were also predicted. These findings provide exploratory biological context for the identified candidate biomarkers and generate hypotheses for future experimental validation.

**Nomenclature**
AKI: acute kidney injury
AMPK: adenosine monophosphate‐activated protein kinase
CaMKII: calcium/calmodulin‐dependent kinase II
CKD: chronic kidney disease
cor: correlation coefficient
DEGs: differentially expressed genes
DNMTs: DNA methyltransferases
ECM: extracellular matrix
ESRD: end‐stage renal disease
GBM: glomerular basement membrane
GEO: Gene Expression Omnibus
GO: Gene Ontology
GSEA: gene set enrichment analysis
IgAN: immunoglobulin A nephropathy
*IL-6*
: interleukin‐6
IRI: ischemia‐reperfusion injury
KEGG: Kyoto Encyclopedia of Genes and Genomes
m6A: N6‐methyladenosine
MAPK: mitogen‐activated protein kinase
miRNAs: microRNAs
ML: machine learning
*MMP-9*
: matrix metalloproteinase‐9
mTOR: mammalian target of rapamycin
NK: natural killer
PI3K: phosphatidylinositol‐3‐kinase
PKC: protein kinase C
Rho: Ras homolog
TFs: transcription factors
*TNF-α*
: tumor necrosis factor‐alpha
VCRGs: vascular calcification‐related genes
*VEGF*
: vascular endothelial growth factor

## Author Contributions

Conceptualization: LiJuan Cheng. Data curation: LiJuan Cheng and ZiJin Xin. Formal analysis: XinLi Zhao. Funding acquisition: LiJuan Cheng. Investigation: JingFeng Wang. Methodology: LiJuan Cheng and ZiJin Xin. Resources: JingFeng Wang. Software: XinLi Zhao. Supervision: ZiJin Xin. Validation: LiJuan Cheng. Visualization: XinLi Zhao. Writing—original draft: LiJuan Cheng. Writing—review and editing: LiJuan Cheng, ZiJin Xin, XinLi Zhao, and JingFeng Wang.

## Funding

This study was supported by the 2023 Nanshan District Science and Technology Plan Project (NS2023039) and the Major projects of Nanshan District′s health system in 2025 (NSZD2025024).

## Disclosure

All authors read and approved the final manuscript.

## Ethics Statement

The authors have nothing to report.

## Consent

The authors have nothing to report.

## Conflicts of Interest

The authors declare no conflicts of interest.

## Supporting Information

Additional supporting information can be found online in the Supporting Information section.

## Supporting information


**Supporting Information 1** Figure S1: Comparison of estimated immune cell proportions between the IgAN and control groups after Benjamini–Hochberg correction.

**Supporting Information 2** Table S1: Summary table of 709 VCRGs.

**Supporting Information 3** Table S2: Summary table of GO analysis of candidate genes.

**Supporting Information 4** Table S3: Summary table of KEGG analysis of candidate genes.

**Supporting Information 5** Table S4: Summary table of GSEA analysis of JUN.

**Supporting Information 6** Table S5: Summary table of GSEA analysis of FOS.

**Supporting Information 7** Table S6: Summary table of GSEA analysis of COL1A2.

## Data Availability

The data that support the findings of this study are openly available in Gene Expression Omnibus (GEO) database at https://www.ncbi.nlm.nih.gov/geo, Reference Numbers GSE93798 and GSE175759.
